# Autoimmune Hepatitis—Immunologically Triggered Liver Pathogenesis—Diagnostic and Therapeutic Strategies

**DOI:** 10.1155/2019/9437043

**Published:** 2019-11-25

**Authors:** Elisabeth Sucher, Robert Sucher, Tanja Gradistanac, Gerald Brandacher, Stefan Schneeberger, Thomas Berg

**Affiliations:** ^1^Division of Hepatology, Clinic and Polyclinic for Gastroenterology, Hepatology, Infectiology, and Pneumology, University Clinic Leipzig, Germany; ^2^Department of Visceral, Transplant, Thoracic and Vascular Surgery, Division of Hepatobiliary Surgery and Visceral Transplant Surgery, University Clinic Leipzig, Germany; ^3^Department of Pathology, University Clinic Leipzig, Germany; ^4^Department of Plastic and Reconstructive Surgery, Vascularized Composite Allotransplantation (VCA) Laboratory, Johns Hopkins University, Baltimore, Maryland, USA; ^5^Department of Visceral, Transplant, and Thoracic Surgery, University Clinic Innsbruck, Tirol, Austria

## Abstract

Autoimmune hepatitis (AIH) is a severe liver disease that arises in genetically predisposed male and female individuals worldwide. Diagnosis of AIH is made clinically applying diagnostic scores; however, the heterotopic disease phenotype often makes a rapid determination of disease challenging. AIH responds favorably to steroids and pharmacologic immunosuppression, and liver transplantation is only necessary in cases with acute liver failure or end-stage liver cirrhosis. Recurrence or development of de novo AIH after transplantation is possible, and treatment is similar to standard AIH therapy. Current experimental investigations of T cell-mediated autoimmune pathways and analysis of changes within the intestinal microbiome might advance our knowledge on the pathogenesis of AIH and trigger a spark of hope for novel therapeutic strategies.

## 1. Introduction

Autoimmune hepatitis (AIH) is a complex immune-mediated liver disease that is diagnosed histologically by interface hepatitis and high serum levels of alanine aminotransferase (ALT), aspartate aminotransferase (AST), and immunoglobulin G (IgG) and the presence of autoantibodies [1]. The initial perception of AIH as a chronic inflammatory liver dysfunction which mainly affects young Caucasian women [2] has been amplified to both sexes of all age groups and all ethnic societies worldwide [3]. AIH can be asymptomatic or present in various forms from subclinical disease to acute liver failure and end-stage liver disease [4].

Specific diagnostic criteria and scoring systems have been established which include analysis of autoantibodies (ANA, SMA, anti-LKM1, and anti SLA), immunoglobulins (IgG), viral markers (IgM anti-HAV, HBsAg, HBV DNA, and HCV RNA) and histological findings [5]. According to the antibody profile, AIH can be divided into two subtypes. The presence of ANAs and or anti-smooth muscle antibodies (SMA) may indicate AIH type 1 (AIH-1), and anti-liver kidney microsomal antibody type one (LKM1) and anti-LKM3 and/or anti-liver cytosol type one antibody (LC1) are disease markers for AIH type 2 (AIH-2) [6].

The exact mechanisms for the immune tolerance breakdown in AIH have not been described yet, but there is growing evidence that a genetic predisposition, molecular mimicry, and an imbalance between effector and regulatory immunity are key pathologic components for disease development. In this context, several lines of evidence support the central role of impaired T cell number and function [1].

The mainstays of AIH therapy are corticosteroids alone or in combination with azathioprine; however, new therapeutic interventions comprising the entire immunosuppressive armamentarium including biologics as well as cellular-based therapies have been proposed [7]. Liver transplantation (LT) can be a life-saving intervention for patients with acute liver failure (ALF) due to acute severe autoimmune hepatitis (AS-AIH) as well as patients with decompensated chronic AIH or hepatocellular carcinoma. Recurrent disease after LT has been reported in up to 10%-50% of patients, and an onset of de novo AIH has also been described for pediatric and adult liver transplant recipients [8].

This paper will mainly focus on the pathogenesis and diagnosis as well as treatment challenges of AIH. Based on our own empiric data and current standards, it furthermore tries to establish a treatment algorithm for patients with acute hepatitis suspected of having an autoimmune involvement.

## 2. Epidemiology

AIH occurs worldwide, with a variable clinical phenotype and a disparity in age-, gender-, ethnicity-, and geography-related incidence and prevalence [9]. Although uncertain, phenotypic variations and changes may in part rely on environmental, infectious, microbial, and genetic factors [10].

The annual incidence of AIH ranges from 0.67 to 2.0 cases per 100.000, and the annual prevalence ranges from 4.0 to 24.5 per 100.000 people depending on the geographical location [11, 12]. A significant increase in disease incidence has been recognized for Spain [13], Denmark [14], and the Netherlands [15] whereas a stable although permanently high incidence has been reported for New Zealand and the Asia-Pacific Area [16, 17]. This geographical escalation and differentiation can vaguely be explained by the “hygiene hypothesis,” which proposes high sanitation standards, lack of microbial exposure, and hence altered microbiome compositions as the underlying cause of increased systemic immune and autoimmune responses within the population [18]. A dysbiosis of the microbiome which is shaped during infancy may also hypothetically be accounted for the different peaks of AIH onset which range from early childhood to mid- and late adulthood in the aforementioned countries. Further possible explanations for changes in peak age of onset may be the emergence of indigenous triggering antigens inducing immune reactivity in the elderly or disappearance of antigens that triggered autoimmune hepatitis in the young [9]. However, recent reports suggest that AIH might have been simply underdiagnosed outside of the age ranges initially described and might have always been uniformly present in all age groups [19, 20]. The female to male ratio is 4 : 1 and even higher (10 : 1) in AIH type 2 [21]. Mortality in AIH is highest during the first year of diagnosis and exceeds almost sixfold the mortality of the general population. The 10-year liver-related mortality which ranges from 6.2% to 10.2% is different in various ethnic subgroups and may be influenced by cultural and socioeconomic factors such as limited access to medical care [14, 22, 23]. Although the risk of carcinogenesis in AIH is lower than that in viral hepatitis, the occurrence rate of hepatocellular carcinoma (HCC) in cirrhotic patients with AIH ranges from 3.3% to 5.1% [17]. This increased incidence might in part be related to the long-standing course of disease. A large Japanese multicenter study reported a cumulative 1-year and 10-year survival rates of 85.4% and 39.4%, respectively, and the mean survival of these patients was reported to be 3.3 years [24]. Treatment options for HCC in AIH cirrhosis include (i) surgical resection and (ii) percutaneous and (iii) transarterial interventions which are roughly applied in thirds each, depending on the liver function, tumor location, and status of the patient.

The fact that AIH differs in occurrence, phenotype, and outcome suggests that several other triggers might exist that have not been described yet. More population-based studies are hence required to further improve management skills, individualize therapy, and enhance outcomes of patients with AIH.

### 2.1. Pathogenesis of AIH

**Figure 1:**
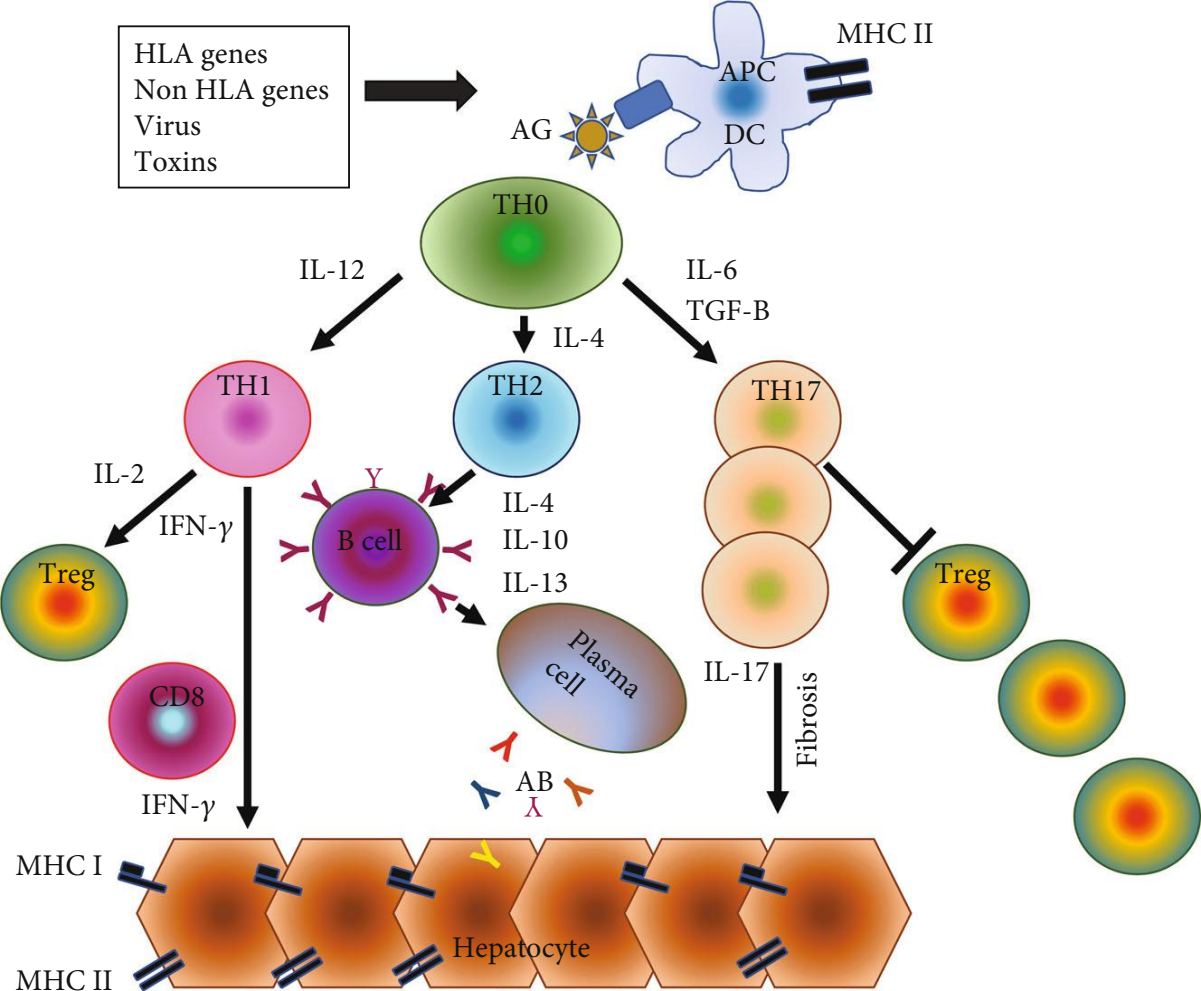
Pathogenesis of autoimmune hepatitis. HLA and non-HLA molecules as well as environmental triggers such as viruses, toxins, and the microbiome have been suggested as key components for a T cell-mediated immune response. The presentation of autoantigenic peptide (AG) to naïve CD4^+^ T helper cells (TH0) by antigen-presenting cells (APC, dendritic cells (DC)) leads to a secretion of proinflammatory cytokines (IL-12, IL-6, and TGF-B) who give rise to the development of Th1, Th2, and TH17 cells. TH1 cells secrete IL-2 and IFN-y, which stimulate CD8^+^ cells to induce expression of HLA class I and HLA class II molecules on hepatocytes. Tregs and Th2 cells secrete IL-4, Il-10, and IL-13 thereby stimulating the maturation of B cells and plasma cells which themselves produce autoantibodies. TH17 cells, which increased number correlates with the degree of liver fibrosis, secrete proinflammatory cytokines and suppress T regulatory cells (Treg). The numerical decrease of Tregs leads to impaired tolerance to autoantigens which subsequently results in the initiation and perpetuation of autoimmune liver damage. The histological characteristics of interface hepatitis comprise an inflammatory cell infiltrate consisting of lymphocytes and plasma cells which is located around the portal tracts.

Although all pathophysiologic mechanisms of AIH are not fully understood, there is growing evidence that a genetic predisposition, molecular mimicry, and an imbalance between effector and regulatory immunity in a particular autoimmune ecosystem are key pathologic factors for disease development [25] (Figure 1). In addition, several other extrahepatic autoimmune disorders have more frequently been described in patients with AIH [26]. It has been widely accepted that a genetic predisposition for autoimmune disease is related to genes within the HLA and non-HLA systems [27].

Although only investigated in small cohorts, susceptibility and resistance to AIH have been associated to DRB1 allelic variants within the HLA region of chromosome 6 [28, 29] and European, American, and Asian studies have described additional geographic variations within HLA alleles associated with AIH [30]. While a predisposition has been associated with the DRB1∗13 or DRB1∗03 and DRB∗07 or DRB1∗03 alleles in South America, Asian studies described an increase in susceptibility of AIH for both DRB1∗0405 and DRB1∗0401 [31, 32]. Particular aggressive courses of diseases have furthermore been attributed to DRB1∗0701 and DRB1∗03-DRB1-04 alleles [33]. In addition, a meta-analysis in the Latin American population revealed protective factors for alleles DRB1∗1302 and DQB1∗0301 [34].

Outside the HLA system, genetic studies identified several single nucleotide variants in the coding regions of tumor necrosis factor-induced protein 3 (TNFIP3) and cytotoxic T-lymphocyte-associated protein 4 (CTLA-4), which have also been associated with the development of autoimmune disease, especially in the Chinese population [35]. A recent meta-analysis on CTLA-4 however only identified one study which showed a positive association of CTLA-4 and AIH [36].

Further putative triggers (e.g., viruses) for AIH have also been linked to the hypothesis of molecular mimicry and cross-reactivity between foreign epitopes and hepatic antigens [3]. This includes hepatitis A virus (HAV) [37], hepatitis C virus (HCV) [38], hepatitis E virus (HEV) [39], measles [40], Epstein-Barr virus (EBV) [41], and herpes simplex virus [42]. Molecular mimicry is furthermore posed as a possible key element for microbiome-associated and drug-induced intestinal autoimmunity. Changes in the composition of the microbiome may lead to increased intestinal permeability, which subsequently facilitates transition of bacteria into the portal circulation [43]. This disruption of the gut barrier axis, which can be influenced by diet and antibiotic exposure, has been demonstrated to facilitate immune-mediated liver inflammation [44]. Pathognomonic changes in gut microbiota have furthermore recently been described in an experimental humanized mouse model of AIH [45]. Further noninvasive gene sequencing and microarray-based biomarker detection within the microbiome of patients with autoimmune disease might hence be of substantial scientific merit [46, 47].

In addition, research has identified several drugs (e.g., minocycline, nitrofurantoin, melatonin, diclofenac, statins, and ornidazole (Table 1)) which may be involved in precipitating AIH. It is important to clarify that drug-induced AIH is a completely different entity from drug-induced liver injury (DILI); however, overlap syndromes have been described in up to 9% of cases in which AIH and DILI are indistinguishable from each other [48]. The assessment of drug-induced AIH is complex; nonetheless, it has been shown that drug metabolites may stimulate the production of autoantibodies by acting antigenic [10]. In this context, priming of the immune system could occur years before apparent disease. Independent from the trigger mechanism, presentation of autoantigenic peptides to CD4^+^ T helper cells (TH0 cells) in general leads to a rise in several TH subsets (TH1, TH2, and TH17) by generation of proinflammatory cytokines, which are moreover involved in complex autoimmune regulations [25]. In this context, experimental data demonstrated that cytokine-activated TH1 and TH17 cells foster an increase in hepatic C-X-C motif chemokine 9 (CXCL9) and C-X-C motif chemokine 10 (CXCL10) expression, thereby triggering progression of AIH in mice [49]. TH17 cells are additionally involved in clearing pathogens during host defense reactions and have been reported to induce tissue inflammation in autoimmune disease through Treg suppression [50]. CXCL10 also known as interferon gamma-induced protein 10 (IP-10), which is secreted by several cell types in response to interferon-Y (IFN-y), has been advocated as a hepatic biomarker of inflammation and fibrosis and has furthermore been proposed as an indicator for disease severity in AIH [51]. On the other side, the divergence of high intrahepatic and low serum levels of Tregs in patients with untreated acute severe AIH [52] could be related to abnormal Treg homing into the inflamed liver, an issue also described in liver transplant patients suffering from acute cellular rejection [53]. In parallel, a decrease in intrahepatic Tregs has been described in AIH patients receiving steroid and azathioprine (AZA) treatment [52] (Figure 1).

### 2.2. Diagnosis of AIH

Diagnosis of AIH is demanding, and in particular, the detection of “acute newly formed” AIH is even more of a challenge [54]. AIH typically manifests as a chronic disease with an insidious onset of chronic liver disease symptoms, and patients may occasionally get diagnosed after incidental discovery of abnormal liver function test [55]. In contrast, up to 20% of patients present with an acute icteric hepatitis which in rare cases may end up with the development of an acute liver failure (ALF) [56]. Acute presentation of disease is generally more common in children, and a subtle onset of disease is vice versa more frequently observed in adults [57].

There is no single pathognomonic test for AIH, and diagnosis is solely based on several indicative clinical, serological, biochemical, and histological findings.

The first diagnostic criteria were established in 1992 by the Autoimmune Hepatitis Group [58] which were subsequently revised in 1999 [59]. The revised criteria however included complex and insufficiently validated parameters of questionable value, which were devised primarily to allow comparison of studies from different centers. In consequence, simplified criteria which enclosed 4 instead of 12 diagnostic parameters were proposed to facilitate a wider applicability in routine clinical practice. These criteria, upon which a score is calculated, include the measurement of autoantibody (titers of antinuclear antibodies ANA, anti-smooth muscle antigen (SMA), and anti-liver-kidney microsomal antibody type 1 (LKM-1)) and immunoglobulin G levels (serum concentrations of globulins or IgG above normal), the evaluation of liver histology (evidence of interface hepatitis, lymphoplasmacytic infiltrate, and rosetting of liver cells), and the exclusion of viral hepatitis (exclusion of viral markers for HAV, HBV, and HCV) (Table 2). A score of 6 is considered as probable AIH and a score of ≥7 as definite AIH [5]. Several data show that the simplified criteria retain a high sensitivity of >80% and a specificity of >95% for the diagnosis of AIH [60].

However, in cases with acute presentation [61] or overlap syndrome [62] where primary biliary cirrhosis (PBC) and primary sclerosing cholangitis (PSC) could coexist in combination with AIH and patients present with clinical, biochemical, serological, and histological features of both a cholestatic liver disease and AIH, diagnostic criteria do not seem to perform so well [63]. However, due to its low frequency and lack of standardized diagnostic criteria, diagnosis of an AIH/PSC or PBC/AIH overlap is complicated. The pathogenesis of overlap syndromes is far from clear, and caution should be used with early diagnosis. Overdiagnosis may lead to unnecessary steroid treatment which in normal cases of PBC and PSC may not be applied. The gold standard for overlap syndrome diagnosis is clinical judgement, and the strongest independent predictor is the microscopic tissue examination of the liver [64].

The prevalence of AIH-PBC overlap is approximately 10% of adult patients, and special “Paris criteria” [65] exist that depending on the presence of at least two out of three key criteria (which for PBC include (i) increased alkaline phosphatase (ALP) or g-glutamyl-transaminase (g-GT) levels, (ii) presence of antimicrobial antibodies (AMA), and (iii) liver histology indicating florid bile duct lesions) provide a clinical diagnosis of an “AIH-PBC overlap syndrome” with high sensitivity (92%) and specificity (97%) [66].

In contrast, diagnostic criteria for the “AIH-PSC overlap,” which has a prevalence of 7%-14%, are less well defined and rely primarily on typical magnetic resonance cholangiopancreatography (MRCP) findings [67].

In the emerging field of IgG4-associated autoimmune diseases, AIH might also be classified into an IgG4-associated and IgG4-nonassociated type, depending on its serological and immunohistochemical presence [68]. Interestingly, the number of T and B cells located in the liver of patients with IgG4-positive AIH is significantly increased, when compared to negative patients. This increased number of liver-specific T and B cell activity has been furthermore linked to better corticosteroid treatment responses in patients with IgG4-associated AIH [69].

In spite of diagnostic criteria, the final diagnosis of AIH needs to be made clinically. Physical examination does not add substantial value to the formation of a solid diagnosis, since symptoms like fatigue, abdominal pain, jaundice, and itching are nonspecific and solely indicate the presence of liver failure. However, AIH should be considered in any patient with acute or chronic liver disease and in particular when increased levels of serum aminotransferases, high immunoglobulins, high titers of circulating antibodies, and other autoimmune diseases are present [70].

### 2.3. Autoantibodies

The measurement of autoantibodies, which has been incorporated in all scoring systems, is a crucial step for both diagnosis and subclassification of AIH and should be performed in all patients suspect for AIH [71]. It is however unclear whether autoantibodies substantially contribute to the pathogenesis of the disease.

Type 1 AIH (AIH-1) which is the predominant type of AIH in both adults and children is characterized by positivity for antinuclear antibody (ANA) and/or anti-smooth muscle antibody (anti-SMA), which can be detected in 80% and 63% of patients, respectively. Concurrent positivity for both antibodies implies a relatively low sensitivity of 43%, with a specificity of 99% and accuracy of 74% [72]. ANAs have shown to be relatively variable markers during the course of AIH, whereas higher titers of SMAs positively correlated with the histologic and biochemical disease activity. This is also true for anti-actin antibodies, which are a subset of SMAs that are present in 86% to 100% of patients with AIH-1 [73]. This means that although SMAs in AIH are mainly directed against filamentous (F) actin, a smaller fraction of 14% has a different molecular target [74].

Type 2 AIH (AIH-2), which only accounts for about 5%-10% of cases, occurs mostly in children and is characterized by the presence of anti-liver/kidney microsomal type 1 (anti-LKM-1) and/or anti-liver cytosol type 1 (anti LC-1) antibody. Incidences of antibodies to LKM 1 are subject to geographical variations. In this regard, they have been detected in up to 38% of pediatric patients in England [75] and only 2% in North American patients [76]. LKM-1 antibodies have a low sensitivity (1%) for AIH; however, their specificity and accuracy are 99% and 57%, respectively [72]. They can furthermore be detected in up to 10% of European patients with hepatitis C virus (HCV) infection [77, 78]. The molecular target of LKM 1 antibodies has been identified to as a short linear sequence in P450IID6 of the cytochrome monooxygenase [79, 80]. In 24% to 32% LC-1 antibodies may appear concurrently with anti-LKM-1 in young European AIH-2 patients [81, 82, 83] and they can also be detected in 12% to 33% of chronic HCV-positive patients [84, 85]. The target antigen has been identified to be a cytosolic enzyme named Formiminotransferase cyclodeaminase [86, 87, 88]. AIH-2 is furthermore characterized to be related with other drug-metabolizing enzymes as autoantigens which also include anti-LKM-2 antibodies directed against CYP2C9-tienilic acid, anti-LKM-3 against UGT1A, and anti-LC1 (liver cytosol antigen-1) and anti-APS (autoimmune polyglandular syndrome type-1) against CYP1A2, CYP2A6, and others [89].

In addition to all aforementioned nonorgan-specific autoantibodies (e.g., ANA, anti-SMA, and anti-LKM-1), several other autoantibodies have been described, which deliver important clues for the diagnosis, disease activity, and prognosis of autoimmune liver disorders [90]. These include the non-liver-specific heterogeneous nuclear ribonucleoprotein (hnRNP) A2/B1 [91, 92] and the liver-specific anti-soluble liver antigen (SLA) for AIH-1 and AIH-2 diagnosis, and positivity for the latter may be associated with a more severe clinical course and worse disease prognosis [93]. It is nowadays accepted that the presence of anti-SLA does not justify the definition of a third subgroup of AIH and should rather allow to classify AIH in type 1 [94].

Since most liver autoantibody titers correlate with age, testing in pediatric patients demands special attention. Contrary to adults, repeated examination in pediatric AIH patients is reasonable, since titers of liver-related antibodies have been shown to correlate with disease activity, and treatment response may be paralleled by the reduction or even disappearance of serum antibodies [95]. Regular autoantibody testing in adults is currently not supported due to the lack of empiric data for antibodies being a surrogate marker for inflammatory disease activity in the older patient.

### 2.4. Liver Histology

In general, liver biopsy is considered a critical element in the differential diagnosis of liver disease and can be weighed as an independent factor to distinguish AIH from other liver illnesses [96]. Furthermore, liver histology is not only a prerequisite for applying the simplified AIH score but is also paramount in both initial diagnosis and long-term follow-up, since it allows for disease staging, therapy monitoring, and assessment for inflammation and fibrosis [3].

Current guidelines recommend liver biopsy at the time of the first presentation [97]; however, in patients with acute liver failure and compromised coagulation status, bleeding risk may be increased. In this context, transjugular or plugged biopsies may be a viable alternative approach in the procurement of a liver specimen for histologic evaluation [98]. A surgical approach to liver biopsy by explorative laparoscopy may be a rather invasive approach which requires general anesthesia.

**Figure 2:**
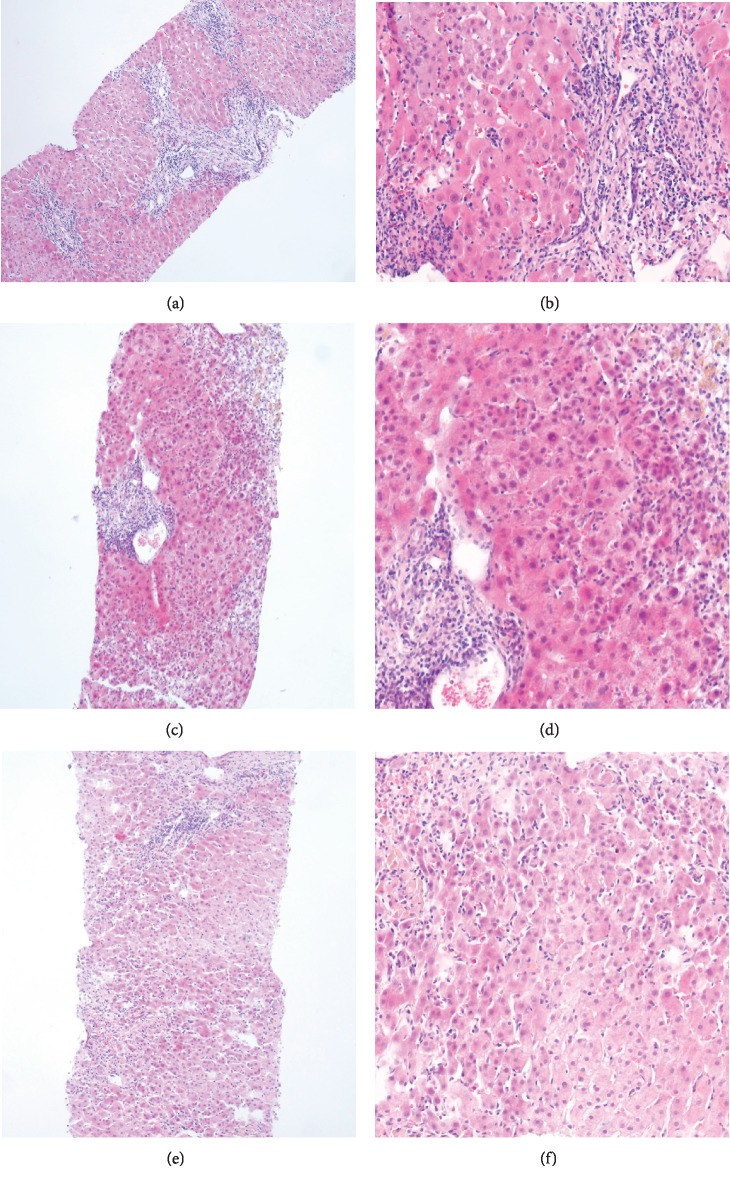
Example of histological features of autoimmune hepatitis (a, b), virus hepatitis C (c, d), and drug-induced liver injury (DILI) (e, f). Haematoxylin and Eosin (H&E) staining: (a, c, e) 20-fold magnification; (b, d, f) 40-fold magnification. (a, b) Interface hepatitis. Liver biopsy histology of a patient with autoimmune hepatitis typically reveals a dense portal and periportal mononuclear cell infiltrate including several plasma cells. The infiltration of lymphocytes and plasma cells in the central and periportal areas reflecting interface hepatitis occurs during the acute phase of AIH but is rather not present during acute hepatitis caused by hepatitis C virus (c, d) and drug-induced liver injury (e, f).

Biopsy findings are laid out to distinguish typical (2 points), compatible (1 point), and atypical (0 points) histologic features of AIH, each contributing variable points to the simplified score. Typical lesions incorporate lymphoplasmacytic infiltrates, presence of rosettes, emperipolesis, and plasma cells, affecting the interface, hence commonly described as interface hepatitis. This nomenclature has been applied due to the apparent sharp contrast between the inflammatory zone and the normal hepatic parenchyma (Figure 2). Interface hepatitis is associated with the development of periportal fibrosis and may progress to bridging fibrosis and ultimately lead to cirrhosis. The accumulation of plasma cells in central areas reportedly occurs during the acute phase of AIH and is not visible in acute hepatitis caused by viruses and drugs.

On the other hand, rosette formation of hepatocytes and emperipolesis is not a “conditio sine qua non” for AIH diagnosis since these changes are commonly seen in other causes of acute lobular hepatitis [99]. Histological examination not only allows for differentiation between AIH and other autoimmune liver diseases (such as primary biliary cirrhosis, primary sclerosing cholangitis, and autoimmune cholangitis); it furthermore allows the diagnosis of up to 20% of AIH patients who do not have detectable autoantibodies. Patients presenting with fulminant hepatic failure histology may solely display massive necrosis and multilobular collapse [100]. Liver histology is not only essential to confirm the diagnosis of AIH; it also helps to assess disease severity and can be instrumental in guiding the intensity of immunosuppressive therapy or estimate the timepoint of setting the patient up for liver transplantation.

## 3. Pharmacological Treatment of AIH

### 3.1. Standard Frontline Treatment: Corticosteroids and Azathioprine

The overall goal of AIH treatment is to induce and maintain complete suppression of the inflammatory activity and to prevent disease progression to cirrhosis and liver decompensation [100]. In this context, treatment can basically be structured into an induction phase and maintenance phase [101]. Remission is achieved when (1) clinical symptoms are absent and (2) transaminases and (3) immunoglobulins have come to normal levels. In children (4), low autoantibody titers are an additional remission criterion.

Standard induction therapy in AIH includes a combination of high-dose prednisolone with or without azathioprine [102]. In case of monotherapy, starting steroid dose is 60 mg/day in adults and 1-2 mg/kg/day in children, not exceeding a maximum dose of 60 mg per day. With regard to combination treatment, differences between the EASL and AASLD guidelines exist, which lie mainly in the starting point of azathioprine which is generally administered at doses of 50 mg daily. While AASLD recommends a simultaneous starting of azathioprine and corticosteroids [103], EASL guidelines also suggest a staggered azathioprine treatment regimen, starting 2 weeks after the introduction of corticosteroids [104]. EASL guidelines furthermore advise that, in patients whom steroid-specific side effects are expected, remission can also be induced by replacing prednisolone with budesonide at a starting dose of 9 mg/day. A recent multicenter randomized controlled trial supported the fact that steroid-specific side effects were less frequent in patients treated with budesonide when compared to those treated with prednisolone. Budesonide furthermore has been shown to induce a higher complete remission rate (reduction to normal ALT) when compared to prednisolone [105].

However, it is important to highlight that budesonide is ineffective in the presence of cirrhosis, excluding at least one-third of AIH patients who have evidence of cirrhosis at timepoint of diagnosis [106]. Furthermore, it is important to stress that in contrast to previous guidelines, where remission was defined by achievement of transaminase levels below twice the upper limit of normal, current guidelines consider normal ALT, bilirubin, and IgG levels as complete remission. This makes the comparison of several retrospective studies difficult since they apparently focus on different study endpoints. Nevertheless, after a successful 4-week induction therapy in which tapering of steroids has already started, depending on the clinical course of the patient (normally at the beginning of week 3), a maintenance phase is initiated with continuous fixed doses of 10 mg of prednisolone and 50 mg of azathioprine daily, until normalization of serum transaminases, bilirubin, or IgG levels is achieved and resolution of histological abnormalities becomes evident [103]. Treatment is usually continued for at least two years [107], and subsequent decision to discontinue therapy generally balances the pros of long-term drug-free remission and cons of relapse-risk need for retreatment [108]. The frequency of achieving a treatment-free state depends on the treatment duration, and recent studies have shown that treatment cessation after 3 to 5 years results in at least temporary remission rates of up to 40% [109]. However, relapse has been shown to be almost universal in patients with autoimmune hepatitis in remission [110].

## 4. Alternative and Second-Line Treatment

### 4.1. Mycofenolate Mofetil (MMF)

Mycofenolate mofetil (MMF) is a next-generation noncompetitive inhibitor of inosine monophosphate dehydrogenase, which acts as the rate-limiting enzyme in purine synthesis. Since, in contrast to other cells, T and B cell proliferation predominantly relies on purine synthesis, MMF is effectively used as an antiproliferative immunosuppressant drug. In solid organ transplantation, MMF has surpassed azathioprine as first-line antirejection therapy and it is currently also used as a frontline or alternative treatment option for autoimmune hepatitis.

In treatment-naïve AIH patients, MMF in combination with prednisolone has been shown to be effective and save in inducing disease remission. A recent meta-analysis comparing standard treatment with MMF and prednisolone also proved MMF to obtain higher remission rates of aminotransferases and IgG levels and lower nonresponse rates [111]. A prospective head-to-head comparison of MMF to azathioprine is still in need; however, corresponding trials are under way (NCT02900443). In patients with corticosteroid-refractory disease or azathioprine intolerance, MMF has also been used successfully as second-line or salvage therapy [4]. On the flipside, MMF has several side effects including gastrointestinal symptoms, and due to its teratogenicity, it cannot be prescribed to a pregnant woman which is highly relevant since AIH predominantly affects women of young age [112]. From an economic standpoint, MMF seems to be 6-7 times more expensive than azathioprine which results in high treatment costs in patients with indefinite treatment length [113].

### 4.2. Calcineurin Inhibitors: Cyclosporine A and Tacrolimus

Cyclosporine A and Tacrolimus belong to the group of calcineurin inhibitors (CNI) which find widespread application as immunosuppressive drugs for solid organ transplant recipients in which scenario they act immunosuppressive by inhibiting Treg activation and IL-2 production [114].

The first clinical data show that Cyclosporine A can effectively be used as frontline therapy of AIH patients, and one small prospective clinical trial at least indicated equivalency to standard AIH therapy [115]. In parallel, Cyclosporine A has also been successfully used as alternative therapy of AIH patients not responding to azathioprine and steroids [116]. Predominantly due to the small number of patients, these studies warrant additional data before Cyclosporine A can confidently be recommended for AIH therapy. Cyclosporine A has been associated with serious side effects including nephrotoxicity, neurotoxicity, infection, and increased incidence of malignancy after long-term use. Animal data also suggest that Cyclosporine A may promote autoimmunity [117, 118] and may have more immunosuppressive capacity than anti-inflammatory activity, both actually inappropriate for AIH treatment.

Although Tacrolimus is a more potent calcineurin inhibitor than Cyclosporine with less nephrotoxic side effects, its use as frontline treatment in AIH is currently not supported by empiric data [119]. This is also due to the fact that the low number of retrospective case series used variable endpoints as remission criteria [120]. Few prospective studies in patients with steroid refractory disease however showed biochemical and histologic improvement with decreased inflammation and reduced fibrosis progression following Tacrolimus treatment [121].

### 4.3. mTOR Inhibitors: Sirolimus and Everolimus

Sirolimus and Everolimus have potent immunosuppressive and antiproliferative properties due to their ability to inhibit the mammalian target of rapamycin (mTOR), a specific intracellular protein kinase regulating cell proliferation, motility, and survival. Both substances have been effectively used in solid organ transplantation [122] and anticancer treatment [123] and on drug-eluting stents in patients with main coronary artery disease [124]. The role of mTOR inhibitors in AIH treatment has to be explored; however, first reports indicate the successful treatment of refractory AIH and recurrent or de novo posttransplant autoimmune hepatitis in a small number of patients [125, 126].

### 4.4. Biologicals: Rituximab and Infliximab

Only recently, monoclonal antibodies, e.g., Rituximab and Infliximab, have effectively been used in patients with refractory or difficult to treat AIH [127, 128, 129] [130].

Although AIH may be considered a T cell-mediated autoimmune disease, Rituximab, which is a monoclonal B cell-depleting antibody, has shown beneficial effects in refractory courses of AIH [131]. One possible explanation for this favorable effect might be the active role of B cells in antigen presentation and T cell suppression which has recently been demonstrated in an animal model of AIH [132]. Clinical side effects of Rituximab comprise infectious complications, which in some circumstances required treatment withdrawal in AIH patients. Although limited, this amount of data clearly supports further investigation of this monoclonal antibody for AIH treatment. This particularly includes the establishment of safety profiles, dosing guidelines, and monitoring strategies [4].

Infliximab is a humanized chimeric monoclonal antibody directed against the proinflammatory cytokine tumor necrosis factor alpha (TNF-a). Since its FDA approval more than 20 years ago, Infliximab has made substantial contribution in the treatment of different chronic autoimmune diseases including AIH [133]. Its clinical use, however, is limited to some small retrospective studies where it was mainly used as a salvage therapy in AIH patients [129, 134]. Infliximab can induce hepatotoxicity-resembling AIH symptoms as well as several other immune-mediated disorders [135]. This is why it should be applied with caution only in specialized centers with a large body of experience in AIH therapy and monitoring.

### 4.5. Thiopurines: Azathioprine (AZA), 6-Mercaptopurine (6-MP), Allopurinol, and 6-Thioguanine (6-TG)

Thiopurines are a group of immunosuppressive drugs that act anti-inflammatory by inhibition of T cell activation and proliferation. Azathioprine (AZA) is a prodrug that is nonenzymatically converted to 6-mercaptopurine (6-MP) which ultimately leads to the generation of 6-thioguanine (6-TG). All three types of thiopurine metabolites can be routinely detected by drug metabolite monitoring and have been extensively used for inflammatory bowel disease (IBD) therapy [136]. In three AIH patients who were unresponsive or intolerant to AZA treatment, 6-MP has also been effective [137]. Another retrospective study on 22 AIH patients with azathioprine intolerance, 6-MP seemed to be beneficial and, in most cases, well tolerated as second-line treatment. Side effects included gastrointestinal symptoms and leukopenia requiring discontinuation of therapy in 23% of patients [138].

Under certain circumstances, AZA treatment can be detrimental due to an altered drug metabolism, which promotes the generation of hepatotoxic metabolites. In this circumstance, coadministration of Allopurinol can redirect thiopurine metabolism back to the physiologic pathway and reestablish effective AIH therapy [139]. Similar effects were recently confirmed in a retrospective case series of AIH patients with skewed thiopurine metabolism, in which Allopurinol was capable to reestablish normal AZA function [140].

6-Thioguanine (6-TG), which is approved for the treatment of acute and chronic myeloid leukemia and chronic lymphatic leukemia, was also evaluated as second-line therapy in AIH patients with failed MMF therapy and intolerance to azathioprine [141]. Although these findings were recently confirmed in a retrospective analysis in AIH patients with intolerance to AZA or 6-MP [142], 6-TG cannot be recommended for AIH treatment without reservations, due to its potential hepatotoxicity.

### 4.6. Ursodeoxycholic Acid (UDCA)

Ursodeoxycholic acid (UDCA) has been widely used as treatment of choice in patients with PBC [143]. Through its choleretic action, UDCA hence reduces the retention of bile acid which in PBC and PSC is caused by a defect in hepatic bile acid excretion [144]. In addition, UDCA has been shown to have immunomodulatory properties [145] and can inhibit immune globulin production in a concentration-dependent manner [146]. In this context, UDCA has shown to be beneficial in patients with AIH allowing additional steroid tapering [147, 148]. A recent study on Japanese patients with a histological and serological mild course of AIH even revealed that UDCA monotherapy can achieve and maintain normalization of ALT levels in 71% [149]. However, this clinical value and the definition of further criteria for UDCA treatment indications must be confirmed in prospective studies.

## 5. Differential Diagnosis of AIH and DILI in Patients with Acute Severe Hepatitis and Liver Failure

Acute liver failure (ALF) is a rare syndrome, characterized by an acute disorder in liver coagulopathy, high serum aminotransferases, and hepatic encephalopathy, which rapidly leads to progressive multiorgan failure with unpredictable complications [150].

AIH typically manifests as a chronic illness; however, up to 20% of patients may present with an acute onset of disease which can be associated with the development of acute liver failure (ALF) [56]. The identification of AIH as the etiology of ALF is key to effective therapeutic intervention, because a delay in diagnosis and initiation of steroid therapy results in poor outcomes [151].

In parallel, drug-induced liver injury which is predominantly caused by acetaminophen overdosing, or other idiosyncratic drug reactions, accounts for 13% to 17% of cases of acute liver failure in both the United States and Europe [152, 153]. Differential diagnosis of AIH and DILI presenting with acute liver failure is extremely complicated and frequently ends up with the unsatisfactory diagnosis of indeterminate acute liver failure.

While corticosteroids have been shown to be beneficial in treating acute severe courses [154] and even acute on chronic courses [155] of AIH, treatment of acute forms of DILI primarily focuses on identifying and withdrawing the offending agent. Administration of N-acetylcysteine (NAC), which is generally recognized as the antidote for acetaminophen overdose, has been also shown to significantly improve outcome of nonacetaminophen-induced acute liver failure [156]. Administration of corticosteroids in patients with DILI and ALF is still under debate. While early studies suggested no benefit or even an adverse effect [157, 158], recent publications prove corticosteroids to be safe in patients with severe DILI [159, 160]. In summary, studies suggest that corticosteroids may increase the risk of infection in patients with ALF for both disease etiologies (AIH and DILI) in the event of liver transplantation [161, 162].

**Figure 3:**
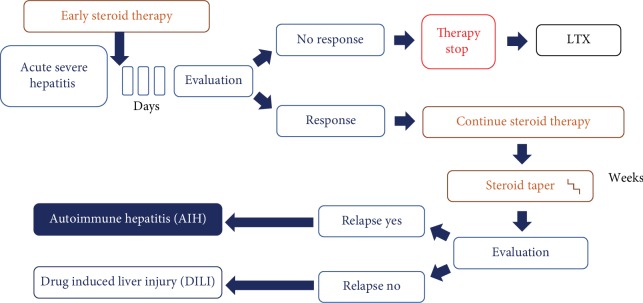
Proposed treatment strategy for indeterminate acute severe hepatitis based on the authors' experience. Patients with diagnosis of acute severe hepatitis receive early steroid treatment. Evaluation after 3 days is aimed at assessing a possible steroid response. If no response is seen, steroid therapy is stopped and alternative treatment strategies are considered. In case of therapy response, steroids are tapered accordingly. After steroid withdrawal, reevaluation may allow for discrimination between autoimmune hepatitis (AIH) and drug-induced liver injury (DILI), since early steroid tapering in AIH is commonly accompanied by a disease relapse.

Several data including our own data suggest that early steroid use in patients with indeterminate acute severe hepatitis is safe and is not accompanied by increased infectious complications. Subsequent dose tapering even allows for early discrimination between AIH and DILI due to specific transaminase flares in patients with AIH [163]. DILI often remains a diagnosis of exclusion. We therefore believe that early and short-term use of corticosteroids is not detrimental but instead beneficial for patients with indeterminate acute severe hepatitis likely developing ALF (Figure 3).

## 6. Liver Transplantation for Autoimmune Hepatitis

Autoimmune liver disease (AILD) represents one of the major indications for liver transplantation and accounts for approximately 24% of transplants performed in Europe and the US [164, 165]. Among the three major AILD entities, namely, AIH, PBC, and PSC, only AIH may present as acute liver failure and hence qualify for high-urgency (HU) liver transplantation [161]. Among AILD, AIH only accounts for a fraction of 3% for pediatric and 5% for adult transplants; nevertheless, in several Scandinavian countries, AILD is the leading indication for liver transplantation, presumably due to a relatively low prevalence of hepatitis C and alcoholic liver disease [166].

In general, transplantation is indicated for patients who present with acute fulminant liver failure which is unresponsive to steroid treatment. Best sets of diagnostic factors associated with outcome for liver transplantation for ALF comprise King's College [167] and Clichy criteria [168, 169]. Further indications include patients with end-stage chronic autoimmune liver disease with a Model of End-Stage Liver Disease (MELD) Score > 15 or higher who have or have not developed HCC. Although there is no single viable predictor for the necessity of LT, patients with a higher MELD score on administration, no improvements in bilirubin and INR levels within the first days of steroid treatment, and presence of necrosis on histology have an increased need for urgent transplantation [170].

Chronic courses of disease with an unacceptable quality of life due to treatment-resistant pruritus or severe hepatic encephalopathy may also benefit from transplantation. HCC occurs in AIH patients with cirrhosis with a variable incidence of 1.9% per year [171]. Accordingly, routine cancer screening and surveillance among this cohort for early detection and treatment is mandatory. The combination of prednisolone and a calcineurin inhibitor is currently the gold standard immunosuppressive treatment regimen for liver transplantation and can also be considered as highly effective in patients with AIH resulting in 1- and 5-year graft survival rates of 84% and 75%, respectively, 5- and 10-year patient survival rates of 90% and 75% [172].

## 7. Recurrent and *De Novo* Autoimmune Hepatitis

In up to 40% autoimmune disease can recur after liver transplantation, despite immunosuppressive therapy [173, 174]. The immunosuppressive regimen however does not seem to have a huge impact on recurrence rate [175]. Acute fulminant AIH is less likely to recur than chronic manifestations. If this is the case, graft dysfunction demonstrates identical features to those of classical AIH [176]. The severity of necroinflammatory activity in the native liver and high IgG levels at the time of transplantation are most reliable predictors of recurrence [176]. Other risk factors for return of AIH after liver transplantation remain unvalidated and controversial [177].

Recurrent AIH is responsive to the reintroduction or dose increase of corticosteroids and azathioprine [178]. Treatment-refractory patients can alternatively be managed with Cyclosporine [125], Sirolimus [179], or MMF [103]. It is important to distinguish recurrent AIH from other etiologies causing liver damage such as rejection, biliary problems, and viral hepatitis [180]. Although uncommon, retransplantation and even return of AIH in a second liver graft have been described [181, 182].

Manifestation of AIH in patients undergoing liver transplantation for other diseases than AIH is called *de novo* AIH [183]. Initially described in pediatric patients [184], de novo AIH has also been reported in adult patients with frequencies ranging from 2.1 to 6.6% [185] and a reported time to development ranging from 0.3 and 7 years post liver transplantation [186]. Although high IgG and autoantibody levels are commonly present in patients with de novo AIH, liver biopsies must be performed to confirm diagnosis [187, 188]. Histology is furthermore an essential element in the differential diagnosis between de novo AIH and graft rejection. The time interval between liver transplantation and onset of disease (de novo AIH) might be an important diagnostic clue [189]. There is also supporting evidence that de novo AIH might be a form of late graft rejection itself [190]. This hypothesis is supported by the fact that antibodies which arise in patients after episodes of acute rejection are directed against graft antigens and not self-antigens. Recurrent courses of rejection have subsequently been described as a risk factor for de novo AIH development [191].

Several studies investigated the genetic background of de novo AIH after liver transplantation and tried to find a relationship to the possession of specific major histocompatibility (MHC) antigens by the recipient and/or the donor [192]. In particular, the status of HLA-DR4 and HLA-DR3 was associated with a risk of recurrence in some research [184, 193]. An increased expression of DRB∗0301 or DRB∗0401 in either donor or recipient has furthermore been described; however, larger numbers of patients are needed to prove the genetic influence on the development of de novo AIH post liver transplantation [186].

Other risk factors associated with the development of de novo AIH include female gender and a donor age of >40 years [194], as well as glutathione S-transferase T1 (GSTT1) donor/recipient mismatch [195, 196].

Recommendations for the treatment of de novo AIH are similar to the standard treatment for recurrent AIH after liver transplantation and comprises the combination of corticosteroids and azathioprine. The majority of cases can be treated effectively, and only a small fraction may progress to graft failure and require retransplantation [197]. Long-term outcomes of de novo AIH have shown excellent results in a study of 31 patients, who reports no death after liver transplantation for de novo AIH after a median follow-up of 7.1 years [198].

## 8. Empiric Data on Diagnosis and Treatment of AIH Patients in Our Own Institution

We investigated the clinical outcome of patients with acute AIH in our single center. A retrospective analysis of data (from 10/2011 to 5/2016) identified 38 patients with eventually newly diagnosed AIH, who presented with clinical symptoms of acute severe hepatitis and high ALT levels (ALT > 5 × ULN, upper limits of normal).

Demographic data are presented in Table 3. Of note, patients had a median MELD score of 16 (range 6-23) at the time of presentation and cirrhosis was evident in 11%. Simplified diagnostic criteria for AIH were available for *n* = 26 patients, revealing definite or probable AIH in 27% and 31% of patients, respectively. 42% had a simplified AIH score of ≤5, indicative for no AIH at the time of presentation. Liver biopsy was performed in *n* = 28 patients (74%), out of which 29% demonstrated typical signs of AIH. However, interface hepatitis was only detectable in 11%.

All patients received tapered corticosteroids and azathioprine with median starting dosages of 60 mg and 100 mg, respectively. AZA was routinely added three weeks after corticosteroid start when tapering of steroid was initiated. 29% of patients were intolerant to AZA, requiring therapy cessation and switch to MMF. In another 5%, Everolimus was added to the MMF therapy plan. Steroid tapering at our institution was performed according to the EASL protocol and guidelines.

**Figure 4:**
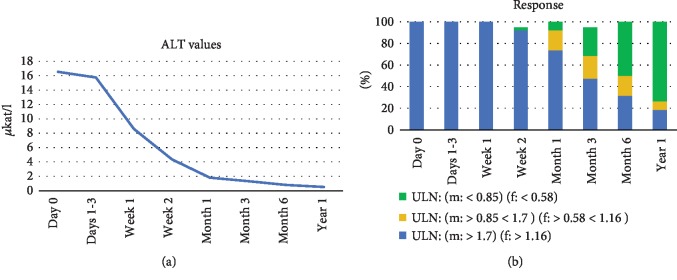
ALT values (a) and response rates in % (b) (indicated as ranges of upper limits of normal, ULN) of AIH patients who received early corticosteroid treatment at our institution. ALT < 0.85 *μ*kat/l for male and <0.58 *μ*kat/l for female patients were considered normal and hence classified as full response (green). ALT levels > 0.85 and <1.7 *μ*kat/l for male and >0.58 *μ*kat/l and <1.16 *μ*kat/l for female were considered as partial response (yellow), and ALT > 1.7 *μ*kat/l for male and >1.16 *μ*kat/l for female were considered as no response (blue).

As depicted in Figure 4(a) , ALT values significantly decreased over time following corticosteroid treatment and in most cases returned to normal values after 6 months. A first full treatment response was detectable in one patient already two weeks after corticosteroid treatment, and response rates significantly increased over time (Figure 4(b)). ALT values reached normal levels in more than 70% of cases after 1 year of medical treatment. In this cohort, probably due to the sufficiently early and effective steroid treatment protocol, we register a 100% survival rate, with no requirement for high-urgency liver transplantation. Infectious complications (3%) were also comparatively low under immunosuppressive therapy in our cohort.

## 9. Conclusion

Over the last decades, substantial progress in understanding the pathogenesis of AIH has been made. In this context, animal models have been vital instruments in detecting different immune cell and cytokine involvement in autoimmune-triggered liver damage. Large human genome studies have identified key predisposing HLA allelic variants associated with AIH development, and several genetic associations outside the HLA locus are under investigation. The currently used AIH scoring systems display acceptable sensitivity and specificity for clinical practice; however, firm diagnostic tools are still in demand. EASL and AASLD guidelines provide a fundamental overview about diagnostic and therapeutic approaches. Corticosteroid treatment with or without azathioprine still remains the gold standard, and liver transplantation is reserved for severe cases presenting with acute liver failure or patients with chronic end-stage liver disease. Novel emerging laboratory techniques may provide a better understanding of the pathogenesis of AIH and facilitate innovative and specific AIH therapy.

## Figures and Tables

**Table 1:** Drugs triggering AIH.

Author	Year published	Drug
Bjornsson et al. [199]	2010	Minocycline
Czaja [10]	2015	Nitrofurantoin
Hong and Riegler [200]	1997	Melatonin
Scully et al. [201]	1993	Diclofenac
Alla et al. [202]	2006	Statins
Kosar et al. [203]	2001	Ornidazole

**Table 2:** Scoring systems for AIH.

Original criteria (minimum req. parameters)	Revised criteria	Simplified criteria
Sensitivity: 85%	Sensitivity: 100%	Sensitivity: 90%
Specificity: 90%	Specificity: 93%	Specificity: 95%
Accuracy: 80%	Accuracy: 82%	Accuracy: 92%
(1) Gender	(1) Female sex	
(2) Serum biochemistry ALP vs. AST	(2) ALP : AST (or ALT) ratio	
(3) Total serum globulin y-globulin or IgG	(3) Serum globulins, IgG	(1) IgG
(4) Autoantibodies	(4) ANA, SMA, LKM-1	(2) ANA, SMA, LKM-1
	(5) AMA	
(5) Hepatitis viral markers	(6) Hepatitis viral markers	(3) Absence of viral hepatitis
	(7) Drug history	
(6) Average alcohol intake	(8) Average alcohol intake	
	(9) Liver histology	(4) Liver histology
(7) Other etiological factors, history of hepatotoxic drug use, or exposure to blood products	(10) Other autoimmune disease	
(8) Genetic factors (other autoimmune disease in patients or first-degree relatives)	(11) Optional additional parameters, HLA-DR3 and HLA-DR4	
	(12) Response to therapy	
Score interpretation Pretreatment: Definite AIH > 15 Probable AIH 10-15 Posttreatment: Definite AIH > 17 Probable AIH 12-17	Score interpretation Pretreatment: Definite AIH > 15 Probable AIH 10-15 Posttreatment: Definite AIH > 17 Probable AIH 12-17	Score interpretation Maximum score: 10 >6: probable AIH >7: definite AIH
*Additional parameters*		
(9) Histology		
(10) Any defined liver autoantibody		
(11) Genetic factors HLA-DR3 and HLA-DR4		
(12) Response to therapy		

**Table 3:** Demographic and descriptive data of AIH patients treated at our institution between 10/2011 and 05/2016.

Demographic data	
Total number 10/2011-05/2016	*n* = 38
Age (median)	50 years
Male	*n* = 16 (42%)
Female	*n* = 22 (58%)
Cirrhosis	*n* = 4 (11%)
Ascites	*n* = 3 (8%)
Hepatic encephalopathy (grade 1)	*n* = 1 (3%)
MELD, median (range)	16 (6-23)
Other autoimmune disease	*n* = 21 (55%)
Simplified AIH score (available for *n* = 26)	
Definite AIH (points ≥ 7)	*n* = 7 (27%)
Probable AIH (points > 6)	*n* = 8 (31%)
No AIH (points ≤ 5)	*n* = 11 (42%)
Liver histology (available for *n* = 28)	
AIH typical	*n* = 8 (29%), interface hepatitis *n* = 3 (11%)
Inconclusive	*n* = 7 (25%)
Not performed	*n* = 10 (36%)
Immunosuppressive therapy	
Prednisolone (mg), median (range)	60 mg (50-100 mg)
Prednisolone therapy duration (days), median (range)	180 days (60-1080 days)
Azathioprine (mg), median (range)	100 mg (50-200 mg)
Azathioprine start after weeks, median (range)	3 weeks (1-12 weeks)
Azathioprine intolerance	*n* = 11 (29%)
Conversion to MMF	*n* = 11 (29%)
Conversion to Everolimus	*n* = 2 (5%)
Outcome	
Survival	*n* = 38 (100%)
Infection	*n* = 1 (3%)
Liver transplantation	*n* = 0 (0%)
